# Early influence of auditory stimuli on upper-limb movements in young human infants: an overview

**DOI:** 10.3389/fpsyg.2014.01043

**Published:** 2014-09-18

**Authors:** Priscilla A. M. Ferronato, Erik Domellöf, Louise Rönnqvist

**Affiliations:** ^1^Department of Pedagogy of Human Movement, São Paulo University, São PauloBrazil; ^2^Physical Education Course, Institute of Health Sciences, Paulista University, São PauloBrazil; ^3^Department of Psychology, Umeå University, UmeåSweden

**Keywords:** review, auditory-motor interaction, upper-limb movements, newborns, infants, development, atypical development

## Abstract

Given that the auditory system is rather well developed at the end of the third trimester of pregnancy, it is likely that couplings between acoustics and motor activity can be integrated as early as at the beginning of postnatal life. The aim of the present mini-review was to summarize and discuss studies on early auditory-motor integration, focusing particularly on upper-limb movements (one of the most crucial means to interact with the environment) in association with auditory stimuli, to develop further understanding of their significance with regard to early infant development. Many studies have investigated the relationship between various infant behaviors (e.g., sucking, visual fixation, head turning) and auditory stimuli, and established that human infants can be observed displaying couplings between action and environmental sensory stimulation already from just after birth, clearly indicating a propensity for intentional behavior. Surprisingly few studies, however, have investigated the associations between upper-limb movements and different auditory stimuli in newborns and young infants, infants born at risk for developmental disorders/delays in particular. Findings from studies of early auditory-motor interaction support that the developing integration of sensory and motor systems is a fundamental part of the process guiding the development of goal-directed action in infancy, of great importance for continued motor, perceptual, and cognitive development. At-risk infants (e.g., those born preterm) may display increasing central auditory processing disorders, negatively affecting early sensory-motor integration, and resulting in long-term consequences on gesturing, language development, and social communication. Consequently, there is a need for more studies on such implications.

## INTRODUCTION

Infants can use multisensory systems from birth (Condon and Sander, 1974; Winkler et al., 2009; Aglioti and Pazzaglia, 2010), utilize environmental information and create new synergies in their action repertoire (e.g., searching behavior with the head and eyes, pre-reaching, and kicking). This developing integration of sensory and motor systems is a fundamental part of the process guiding the development of goal-directed action in infancy. Perception-action studies from birth onward are of critical importance for increased understanding of the developmental processes that underlie the interrelated human motor- and socio-cognitive development. Further, in the case of atypical development, such knowledge is important for early detection of sensory deviances and maturational delays, and for developing effective test protocols and appropriate intervention programs. Whilst the majority of studies of coupling between perception and action in infants have used essentially visual or multimodal perception paradigms, some studies have also found couplings between infant body movements and auditory stimuli (Birns et al., 1965; Condon and Sander, 1974; Fogel and Hannan, 1985; Fernald, 1989; Masataka, 1995; Ejiri and Masataka, 2001; Phillips-Silver and Trainor, 2007). During the past decades, most studies of typical upper-limb movement development have focused on the developmental processes of intentional, object-oriented (i.e., pre-, reaching/grasping) and/or social-oriented communicative behaviors (i.e., gestures/pointing) pre-dominantly in infants from 4 to 5 months of age and onward. Others, mainly from a clinical perspective, have extensively studied spontaneous movement patterns (in fetuses, neonates, and young infants) referred to as general movements (GMs). GMs comprised of a series of gross movements of variable speed and amplitude that involve all body parts. Developmentally, GMs display age-specific characteristics, including so-called “preterm,” “writhing,” and “fidgety” arm movements (Hadders-Algra and Prechtl, 1992). It has been found that the quality and appearance of abnormal GMs are associated with an increased risk of minor neurological dysfunction (Hadders-Algra and Groothuis, 1999). In infants born preterm, delays in the development of reaching and grasping are found to be more common (Thun-Hohenstein and Largo, 1991) as well as reaches with non-optimal kinematic characteristics at the ages of 6–12 months (Fallang and Saugstad, 2003; Rönnqvist and Domellöf, 2006; Grönqvist et al., 2011). Still, although many scholars in the past have pinpointed the evolutionary importance of auditory-motor interactions for human development of gesturing, language and social interaction, few have included the influences of sounds when investigating upper-limb-movements and/or GMs in neonates and young infants, and more recent studies of early auditory-motor interactions are surprisingly rare. Given that the auditory system is rather well developed at the end of the third trimester of pregnancy, it is likely that couplings between acoustics and motor activity can be integrated as early as at the beginning of postnatal life and help motivate environment interaction. Consequently, the study of auditory-motor interactions in infancy stands out as highly relevant from both a theoretical and a clinical perspective.

This mini-review aims to summarize and discuss the emergence of auditory-motor interaction and coordination in typically developing newborns and young infants up to 6 months-old, specifically with regard to studies covering behaviors in the upper-extremities in association with auditory stimuli. A specific focus on the upper-extremities was selected as the acquisition of abilities in those limbs during motor development is of great importance for further possibilities for action in, and interaction with, the environment. The purpose is to present some of the research field on early active behavior and auditory-motor integration, and to bring further understanding for its significance for the developmental process during early infancy. Another purpose is to highlight the need for further studies focusing on infants at risk for developmental delays and deviations related to early auditory-motor interactions that may lead to negative long-term consequences.

## ACTIVE BEHAVIOR

Even a simple movement involves a complex synergy between action, perception, and cognition in order to understand, construct, and produce a proficient action to successfully achieve the intended goal. Already from early stage development, human infants perform prospective actions following these principles (von Hofsten and Rönnqvist, 1988; Claxton et al., 2003). In newborns, simple actions such as head turning or single displacements of the limbs from one position to another are characterized by continuous velocity profiles, produced by a regular pattern of accelerations/decelerations (von Hofsten, 1993; von Hofsten and Rönnqvist, 1993; Lee, 2006). Still, relatively little research has been devoted to the coupling between motor action and various sensory stimuli in young infants when interacting with the environment. Several studies, however, suggest that early motor outputs are indeed to be seen as potentially intentional and controlled behaviors in interaction with environmental events, including sucking behavior (Kalnins and Bruner, 1973; DeCasper and Fifer, 1980; Walton et al., 1992; Rochat and Hespos, 1997; Bobin-Bègue et al., 2006), movement of the neck and head (Jouen, 1988), and leg movements (Thelen, 1981).

Active behavior has also been studied and identified with regard to arm movements displayed by healthy newborn infants (van der Meer et al., 1995; van der Meer, 1997). These findings provide support for an early focused control of arm movements guided by vision. With regard to reaching, and grasping behavior, there is currently no consensus in the literature about when newborns are able to couple arm and hand movements to environmental events and display intentional reaching action. Several studies have reliably confirmed intentional behavior during reaching and grasping in infants around 7–8-months-old (Lockman et al., 1984; von Hofsten and Fazel-Zandy, 1984; Pieraut-Le Bonniec, 1985; von Hofsten and Rönnqvist, 1988; Corbetta et al., 2000; Fagard, 2000). Other studies report similar intentionality in infants at around 4 months, as observed in prospective grasp-planning ability (Newell et al., 1989; Siddiqui, 1995; Barrett et al., 2008). In agreement with the latter reports, infants at 4 months display modulation of hand pressure relative to an object’s rigidity (Rochat, 1983). Further, infants at 4 months appear sensitive to the position of objects when planning to grasp (Bruner and Koslowski, 1972; McDonnell, 1975). However, indications of intentional reaching have been noted even earlier. Another study observed oriented reaching toward seen objects in newborns, interpreted as an indication of intention (Bower et al., 1970). Trevarthen employed the term “pre-reaching” when observing a precise coordination between hands and fingers in infants’ attempts to reach at around 2 months (Trevarthen, 1974). Pre-reaching was later longitudinally evaluated in newborns and infants over the ages 5–16 weeks showing that a reaching reaction directed to the object could be observed at 5 weeks, becoming increasingly intentional and active over the subsequent weeks (von Hofsten, 1984).

To summarize, the literature shows evidence that active behavior is observable from the very beginning of life and over early infancy, and couplings between perception and action are undoubtedly involved already in newborns’ motor behavior.

## AUDITORY STIMULI AND BODY-BEHAVIOR INTERACTION

Research on cross-modal perception in newborns and young infants has established an early coordination of hearing with other sense modalities (Wertheimer, 1961; Turkewitz et al., 1966; Bower et al., 1970; Mendelson and Hath, 1976; Crassini and Broerse, 1980; Fenwick and Morrongiello, 1998; Morrongiello et al., 1998). Most developmental perception-action studies have employed parallel intermodal matching of auditory-visual relationships when investigating early infant behaviors (Guihou and Vauclair, 2008). Few have studied the role of amodal sound information in relation to newborn motor activity and in coordination with the environment. Auditory experience begins at the start of the third trimester of pregnancy as the myelination process from the cochlear outlet up to the auditory thalamus occurs (Moore and Linthicum, 2007). As shown by ultrasound studies, fetal facial and body movements in response to acoustic stimulation can be observed from 25 to 27th gestational weeks and with consistency during the following gestational weeks (Birnholz and Benacerraf, 1983; Kuhlman et al., 1988; Hepper and Shahidullah, 1994). The auditory system is therefore fairly developed at birth, but still immature during early infancy in terms of, e.g., restricted sensitivity to high-frequency sounds (Werner, 2007). This means that newborns and young infants (and fetuses) are more sensitive to low-frequency sounds such as speech and language (Hepper and Shahidullah, 1994). Indeed, studies have shown that fetuses display behavioral changes (heart rate deceleration) when exposed to low-pitch auditory stimuli (Lecanuet et al., 2000), may discriminate between male and female voices (Lecanuet et al., 1993), and that fetuses and newborns recognize the mother’s voice after prenatal exposure (DeCasper and Fifer, 1980; Smith et al., 2007; Kisilevsky and Hains, 2011). This also implicates that speech stimuli may evoke different responses compared with other auditory stimuli that may be valuable to consider when investigating couplings between acoustics and motor activity in early postnatal life.

Studies of newborns and young infants have shown a relationship between various behaviors such as head turning, sucking, visual fixation, and auditory stimuli. The outcomes of these studies concurrently suggest that infants are able to change behaviors according to different sound conditions. For instance, newborns, and young infants are able to turn their heads toward a sound source (Chun et al., 1960; Siqueland and Lipsitt, 1966; Clifton et al., 1972; Muir and Field, 1979; Morrongiello and Fenwick, 1994). The most common explanation for this phenomenon is that newborns are born with some mechanism specific for turning to sound or for sound localization as a kind of innate reaction ability. None of these studies, however, have investigated or discussed the potential active components involved in head turning to sound (i.e., intentionally turning the head based on auditory information).

In studies comparing newborn sucking behavior and auditory stimuli, it has also been observed that sucking can vary according to auditory stimulation (Keen, 1964; Lipsitt and Kay, 1964). Active sucking in newborns has also been reported in association with a learning task during contingent or non-contingent presentation of speech sounds (Floccia et al., 1997), suggesting learning abilities through sucking behavior in operant conditioning. Moreover, study conditions involving speech sounds and/or where auditory stimuli is presented in a social context (e.g., mother’s face in view and conversational interaction) seem to promote active sucking behavior in newborns (DeCasper and Fifer, 1980). Newborns and infants also move their eyes according to side of sound presentation (Turkewitz et al., 1966; Hammer and Turkewitz, 1975). Again, however, the discussion is based on innate components of turning to sound, with the young infant having a mainly passive role in the observed phenomenon.

In summary, according to most of the aforementioned studies, head, and facial movements of newborns and young infants seem to be displayed differently in association with auditory stimuli. However, few studies have considered that such behaviors can be used actively to interact with the environment in different auditory conditions, perhaps because it is not the most obvious function of this body region. Still, there seems to be some evidence of an early ability to be actively involved in exploratory activity and that newborns and young infants can perform perception-action sequences with different parts of the body according to acoustic conditions. In support of such statement, one study explored infant leg movements in relation to auditory stimuli (Stanley and Madsen, 1990), showing that infants (2–8 months old) could actively change auditory stimuli by changing their kicking behavior. The mother’s voice was most commonly preferred, followed by a female voice, and then music. Infants learned how to change the acoustic stimuli and changed more often from non-preferred to preferred acoustic conditions.

## AUDITORY STIMULI AND UPPER-BODY BEHAVIOR INTERACTION

A systematic search for English-language articles devoted to relation between auditory stimuli and upper-limb movements in infants up to 6 months between 1960 and 2014 was performed. A total of 18 articles were identified, derived from one or more of the following electronic databases: EBSCO, PubMed, Web of Science, Science Direct, OvidSP, Research Gate, and Google Scholar. The search terms used were a combination of: “auditory stimuli,” “sound information,” “arms movement,” “arms behavior,” “upper body movement,” “manual behavior,” “hand movement,” “hand behavior,” “newborn,” “young infant,” “infant.” Additionally, the search was supplemented by exploring the reference list of included publications (see **Table 1**).

**Table 1:** Studies of early auditory-motor interaction in newborns and infants up to 6 months included in the review.

Author	Participants	Motor behavior	Method	Auditory stimuli	Results
Birns et al. (1965)	20 newborns; age range: 36–96 h	Body movements	12 trials; online rating of behavioral excitation	Continuous tone (85/90 db) vs. intermittent tone (85 db) vs. silence	The (aroused) newborns were differentially soothed by auditory stimuli. Greater decrease of activity and level of arousal for low-pitch than high-pitch tone
Condon and Sander (1974)	16 newborns; age range: 12 h-14 days	Body movements	1 session; frame-by-frame analysis (30 frames/s)	Male voice (talking)	Synchrony between auditory stimuli and body movements
Collyer and Bench (1974)	11 newborns; age range: 2–8 days	Body movements	Video recordings during 6 trials (3/ear); coding of behavior/activity state	Sound (70 db) during 5 s vs. during 60 s vs. silence	Higher activity states at the onset of sound than in the silence conditions
Thelen (1979)	20 infants; age range: 28–52 weeks	Body movements	Observation of body movements	Babble	Peak in rhythmic arm activity at the same age when they begin to babble (4 months)
Kato et al. (1983)	32 newborns; age range: 1–6 days	Body movements	30 h video recording of leg/arm movements (73 selected periods)	Human voice (spoken to) vs. non-human sound (noise)	Body movement reaction to human voice but not to non-human sound. Newborns can correlate movements with different voices
Miller and Byrne (1983)	24 newborns; age range: 30 72 h	Body movements	Heart rate measurement; coding of behavioral state and body activity during light sleeping	Sound varying in: (1) duration of the transition and (2) pulsed vs. continuous	Heart rate increased but no significant differences in body activity as an effect of auditory stimuli
Rönnqvist and von Hofsten (1994)	26 newborns; age range: 2–6 days	Arm-, hand-, finger movements	Video recording, three conditions: baseline (no stimuli), social (face to face with mother), object (ball moving in front of newborn)	Talking interaction with mother (social condition) and rattle sound attached to the object (object condition)	Different movement patterns between conditions. More finger movements, transitional, and flexion movements of hand in the social condition and more thumb-index finger activity and whole hand extension in the object condition
Locke et al. (1995)	61 infants average age from 18.4 to 37.5 weeks-old	Manual activity	Audio and video recording	Audible or inaudible rattles	Increased shaking in infants who had begun to babble
Ejiri (1998)	28 infants; age range: 5–9 months	Arms shaking	Video recording	Audible or inaudible rattles	Rhythmic activities reached their peak around the onset of canonical babbling
Dibiasi and Einspieler (2002)	29 infants; age range: 9–14 weeks	Body movements	Video recordings of spontaneous movements	Sound (68/77/88db) vs. silence	Tendency to decrease/stop movements in the acoustic (88dB) compared to the silence condition
Iverson and Fagan (2004)	47 infants from 6 to 9 months-old	Body movements	Video recordings during semi-structured play session	Vocalization	Manual activities are linked with communicative gesture-speech system
Nakata and Trehub (2004)	43 infants; age range: 5.5–6.5 months	Body movements	Audiovisual presentation (4 min segments); video recording; coding of minimal body movement	Mother talking vs. mother singing	Reduction of body movements shown more often in the mother singing than mother talking condition
Iverson et al. (2007)	26 infants; age range: 4–7 months	Arms shaking and laterality biases	Play with infants rattles (video recorded)	Rattle with and without sound	Rate of rattle shaking increased from the pre-babble to the babble onset session
Iverson (2010)	NA	NA	Literature review	NA	Motor development is participatory in the emergence of language
Zentner and Eerola (2010)	120 infants; age range: 5–24 months	Body movements	Video recordings and kinematic recordings of body movements; coding of body movement engagement	Eight music/rhythm stimuli: two excerpts of classical music, rhythm-only version of classical music excerpts, children’s music, isochronous drumbeats, music stimulus with rapid tempo shifts vs. two control stimuli: adult-directed speech and infant-directed speech (65 db)	More rhythmic movements to music/rhythm than speech, some tempo flexibility, and more rhythmic coordination with displays of positive affect. Youngest infants (5–7 months) less consistent in behavior than older (less overall rhythmic movements and as much movement to infant-directed speech as to music/rhythm stimuli)
Morgan et al. (2013)	51 infants; age range: 4–7 months	Body movements	Video recording during 1 (10 min) session; coding of level of body activity	Classical music and movie vs. classical music and static image	All infants displayed body movements with music (but not rhythmic movements). More movements in the static image than movie condition
van der Meer and van der Weel (2011)	NA; age range: 3–6 weeks	Arm movements	Sound presentation from speakers attached to right/left wrist; measurement of distance between ipsilateral wrist and ear	Mother talking	Decreased distance between the wrist and ear at the side of sound presentation, indicating intentional control of arm
Lee and Newell (2013)	11 infants; age range: 10–16 weeks	Arm movements	Biweekly observations; 6 trials (60 s); kinematic recordings of right arm-hand movements	Mother’s voice vs. musical tones vs. silence	Both auditory stimuli conditions increased amplitude of arm movements and number of reaches compared with the silence condition
Fujii et al. (2014)	30 infants; age range: 3–4 months	Limb movements and vocalization	Video recordings and kinematic recordings of limb movements with simultaneous sound recordings of vocalization	Music (beats) or silence	Limb movements and vocalization synchronized to the music

*NA, information not available; db, decibel; s, seconds; ms, milliseconds; EMG, electromyography.*

Undeniably, evidence for active use of the upper-extremities seems to be present from early infancy. For instance, one study showed that newborns at 2–6 days old performed different movement configurations with the arms, hands, and fingers when alternated between social, object presentation, and baseline conditions (Rönnqvist and von Hofsten, 1994). Analyses of the performed movements were in agreement with condition, showing more finger movements and flexions of the hands in the social condition, and more thumb-index finger activity and extension of the arms and hands in the object condition. Of relevance to the present review, mothers talked to their infants in the social condition (auditory stimulation available), and a small rattle was attached to the target object to initiate attention to the object. Thus, as in most studies of auditory-movement interaction in newborn/young infants striving for high ecological validity, other sources of stimulation were simultaneously available (e.g., visual stimuli).

Other studies have investigated whole body movements (including arm movements but not analyzed separately) in different auditory conditions (Collyer and Bench, 1974). Despite some inconsistencies between findings (Birns et al., 1965), likely due to methodological differences, these studies generally suggest that body movements, including arm movements, can be expressed in different ways according to various auditory stimulation from the beginning of life, as well as demonstrating acoustic preferences. Indeed, studies of newborn and infant body rhythm engagement in association with sound stimuli seem to confirm an early ability for bodily engagement with sound information in the environment (Condon and Sander, 1974; Zentner and Eerola, 2010). Additionally, in a recent study it was found that infants at 3–4-months-old interacted with music via limb movements and vocalizations, suggesting that the human brain is primed with the body to interact with music already at this age (Fujii et al., 2014).

Rhythmic arm movements in infants has gained a particular research interest in relation to the onset of reduplicated babble. At about 4-months-old, infants show a peak in rhythmic arm activity such as hand banging (Thelen, 1979), temporally coinciding with the age when infants begin to babble. The performance of rhythmic arm movements share common grounds with reduplicated babble in terms of producing organized and timed actions, and may provide multisensory feedback (including auditory from the infants’ own sound production) that works in support of language development (Iverson, 2010). Other studies showed an increase in infant rhythmic arm activity when leaving the pre-babbling age to onset of babbling, and a decline when becoming experienced babblers (Locke et al., 1995; Ejiri, 1998; Iverson et al., 2007; Iverson, 2010). Moreover, as babbling begins, vocal, and manual activities may be further linked and coordinated, underlying a later communicative gesture-speech system (Iverson and Fagan, 2004; Iverson et al., 2007). Thus, infant arm movements and vocalizations seem to be interrelated skills of importance to the acquisition of language.

Although specific investigations of arm movements in relation to auditory stimuli in younger infants are rare, one study observed that newborns from 3 to 6 weeks could control their arms to listen to the mother’s voice according to the side of sound presentation (van der Meer and van der Weel, 2011). Mini-speakers were attached to the newborns’ right and left wrists and the mother’s voice was presented in either speaker. Measuring the distance between the ipsilateral wrist and ear, it was found that the distance between the wrist and ear decreased at the same side as the sound was presented. Recently, another study recorded the amplitude of arm movements to investigate if different auditory conditions (mother’s voice, music, and silence) affected the reaching behavior in infants from 10 to 16-weeks-old. The results showed that the amplitude of exploratory arm movements prior to reaching and the number of reaches increased in the mother’s voice and music conditions compared with the silence condition (Lee and Newell, 2013). Taken together, these findings suggest that infants older than 3 weeks can integrate auditory information and display different arm movements depending on different sound conditions. The findings further suggest that infants are capable of using auditory information to explore action opportunities before executing goal-directed arm movements. It may thus be that multisensory connections between the auditory system and the motor system are underlying self-initiated spontaneous arms movements from very early in human life. Again, however, it must be considered that auditory stimulus was not the only information source to guide the motor system in these studies. Beyond the auditory information, visual information was also available, and thus, in parallel with the proprioceptive and haptic information, contributed to the organization of the infants’ upper-body movements.

## DISCUSSION

The general summation of this overview is that human newborns and young infants appear able to (1) link motor actions with sensory stimulation and perform goal-directed and prospective movements that are not simply reactive, (2) change body movements according to different auditory conditions, also in an active, explorative or preferential manner, and (3) at least from 3-weeks-old, integrate auditory information and perform diverse arm movements depending on different sound conditions, possibly as a means of exploring action opportunities. However, the literature concerning auditory-motor interactions in young infants, particularly regarding upper-limb movements, and those that reflect the outcome of abnormal and/or delayed audio-manual-action development, is still limited.

The importance of advancing this research area becomes clear in the perspective of atypical development. Significant hearing loss is described as one of the most common disorders at birth, occurring in 1–2 per 1000 newborns (Korver et al., 2010) increasing the risk for delayed language development, difficulties with behavior and psychosocial interactions, and poor academic achievement. Infants born preterm and/or with very low birth weight are also at increased risk for hearing loss, associated with central auditory processing disorders that hamper ability to discriminate simple speech sounds and limit auditory memory span (Davis et al., 2001). Alternatively, they may experience progressive or delayed-onset hearing loss (Cristobal and Oghalai, 2008). These difficulties may have a negative effect on early sensory-motor integration, gesturing and social communication, and the acquisition and development of language skills and learning. Thus, further longitudinal studies, starting from an early postnatal age, to investigate the effect on auditory-movement interaction in at-risk infants are needed. It is of high importance to discover delayed auditive maturation and/or immature auditory pathways early (screening for hearing loss in the newborn allowing for earlier interventions and relevant hearing aids), as such conditions may lead to both short- and long-term consequences for auditory-movement interaction and coordination, with implications for further social and cognitive development.

## CONCLUSION

The relationships between auditory (sensory) information and motor execution have an essential function for the integration of and adaptation between internal and external information in human newborns and young infants, and play a significant role for the developmental process of social interaction and communication, language acquisition, and further cognitive performance. Whilst several important observations of auditory-motor interactions in infancy have been made, lately also with regard to upper-limb movements, more studies are warranted. In this effort, a particular focus should be put on the effects/developmental processes in infants at risk for developmental delays and/or sensory deviations affecting multisensory and motor integration from an early age onward.

## AUTHOR CONTRIBUTIONS

Priscilla A. M. Ferronato was in charge of the primary literature search and selection of relevant articles, contributed in the summation, integration and discussion, prepared the first draft of the paper, and approved the final draft as submitted. Erik Domellöf contributed in the selection of relevant articles, contributed in the summation, integration and discussion, co-wrote, reviewed, and revised the manuscript, and approved the final draft as submitted. Louise Rönnqvist contributed in the selection of relevant articles, contributed in the summation, integration, and discussion, co-wrote, reviewed, and revised the manuscript, and approved the final draft as submitted.

## Conflict of Interest Statement

The authors declare that the research was conducted in the absence of any commercial or financial relationships that could be construed as a potential conflict of interest.
